# Breathing-Practice Status and Psychological Outcomes 12 Months After a Structured Program in University Students

**DOI:** 10.3390/bs16101745

**Published:** 2026-09-24

**Authors:** Alexandru Florian Crișan, Roxana Ramona Onofrei, Adelina Marițescu, Cristian Oancea, Elena Amaricai, Daniela Șilindean

**Affiliations:** 1 Research Center for Assessment of Human Motion, Functionality, and Disability, Victor Babeș University of Medicine and Pharmacy Timișoara, Eftimie Murgu Square 2, 300041 Timișoara, Romania; crisan@umft.ro (A.F.C.); amaricai.elena@umft.ro (E.A.); 2 Victor Babeș University of Medicine and Pharmacy Timișoara, Eftimie Murgu Square 2, 300041 Timișoara, Romania; adelina.maritescu@umft.ro; 3 Center for Research and Innovation in Personalized Medicine of Respiratory Diseases (CRIPMRD), Victor Babeș University of Medicine and Pharmacy Timișoara, Eftimie Murgu Square 2, 300041 Timișoara, Romania; oancea@umft.ro; 4 Center for Applied Linguistics and Comparative Cultural Studies (C-CLASC), Victor Babeș University of Medicine and Pharmacy Timișoara, Eftimie Murgu Square 2, 300041 Timișoara, Romania; silindean.daniela@umft.ro

**Keywords:** breathing practice, positive affect, sleep quality, life satisfaction, student well-being, practice maintenance

## Abstract

Evidence regarding long-term breathing-practice maintenance and associated psychological outcomes remains limited. This observational longitudinal follow-up examined psychological outcomes and self-reported breathing-practice status 12 months after a structured program in university students. Thirty-four students from the intervention group of a 14-week breathing study were assessed immediately after the program (T1) and at the 12-month follow-up (T2). At T2, 18 reported occasional or regular practice, and 16 reported no or rare practice. All 13 psychological outcomes were included in the longitudinal analyses. For each outcome, the T2 score was regressed on the corresponding T1 score and T2 practice group using HC3 robust standard errors. Benjamini–Hochberg correction was applied across all 13 outcomes. Compared with the no/rare-practice group, the ongoing-practice group had more favorable T2 scores for subjective happiness, general happiness, positive affect, sleep quality, all six dimensions of psychological well-being, and life satisfaction after adjustment for the corresponding T1 scores; all q < 0.001. However, the ongoing-practice group also had higher worry scores (adjusted difference 4.52, 95% CI 1.44 to 7.60; q = 0.006). Negative affect did not differ between groups (−0.50, 95% CI −3.51 to 2.50; q = 0.735). Sensitivity analyses produced the same pattern. Ongoing breathing practice was associated with several favorable psychological outcomes at 12 months, but also with higher worry scores and no difference in negative affect. Because practice status was self-selected and measured concurrently with the outcomes, these findings are associative rather than causal and may be influenced by common-method bias, recall bias, and social-desirability bias.

## 1. Introduction

Breathing exercises are increasingly recognized as simple and affordable approaches that may influence physiological processes, such as autonomic regulation and respiratory efficiency, as well as psychological outcomes, including stress, mood, and emotional stability (Zaccaro et al., 2018).

Short-term interventions, such as structured breathing programs lasting 6 to 16 weeks, have reported small-to-moderate improvements in psychological outcomes, including reduced anxiety, improved sleep quality, and increased positive affect (Fincham et al., 2023; Jerath et al., 2015; Ma et al., 2017).

Although some breathing-intervention studies have included follow-up assessments, these generally cover shorter periods and rarely examine whether participants continue practicing independently after the structured program (Balban et al., 2023; Fincham et al., 2023; Steinmane & Fernate, 2025). Evidence combining a 12-month psychological assessment with detailed information on practice maintenance, frequency, perceived benefits, barriers, and adverse effects remains limited.

Recent studies show that university students are experiencing increasing psychological pressures: for example, in the last year, over one-third of students reported moderate to severe anxiety, and more than half reported significant stress or depressive symptoms (Ahmed et al., 2023; Gambolò et al., 2025; Tan et al., 2023). Beyond immediate outcomes, the success of such interventions ultimately relies on whether individuals incorporate the practices into their daily routines.

The aim of this exploratory follow-up was to characterize breathing-practice patterns at 12 months and examine whether self-reported practice status at T2 was associated with each of the 13 psychological outcomes assessed at T2, after adjustment for the corresponding T1 score.

## 2. Materials and Methods

### 2.1. Study Design

This exploratory longitudinal follow-up included participants from the intervention group of a previously published non-randomized controlled study (Crișan et al., 2025). Psychological outcomes were assessed at three time points: before the 14-week breathing program (T0), immediately after completion of the program (T1), and 12 months after program completion (T2). The original control group completed T0 and T1 assessments but had no corresponding T2 data and was therefore not included in the present longitudinal analysis. No additional sample-size calculation was performed because the eligible sample was fixed by the size of the original intervention cohort.

The study was conducted at the Victor Babeș University of Medicine and Pharmacy Timișoara within the undergraduate physiotherapy program. The original program comprised one supervised 30-min small-group session per week for 14 weeks, with 6–10 students per group. Sessions were delivered by a physical therapist specialized in respiratory therapy and included brief theoretical instruction, practical demonstration, supervised practice, and individualized correction. Approximately 15 min were allocated to diaphragmatic breathing, 5 min to pursed-lip breathing, and 5 min to alternate-nostril breathing; the remaining session time was used for instruction and feedback.

Diaphragmatic breathing involved placing one hand on the abdomen and the other on the chest, directing the breath toward the diaphragm, and minimizing upper-chest movement. During pursed-lip breathing, participants inhaled through the nose and exhaled slowly through pursed lips. Alternate-nostril breathing was performed by closing each nostril sequentially with the thumb and ring finger, inhaling through one nostril and exhaling through the other. These techniques were selected because they were simple, required no equipment, and represented complementary breathing-control strategies. The sequence progressed from basic diaphragmatic awareness to prolonged expiration and then to the more coordination-demanding alternate-nostril technique (Crișan et al., 2025).

No fixed respiratory rate or standardized inspiratory-to-expiratory phase duration was prescribed or recorded. Participants practiced at a comfortable pace while following the technique-specific instructions. Daily home practice was encouraged through written guidance and personalized recommendations, but the frequency, duration, and fidelity of practice were not objectively monitored during the original program.

The original 14-week study received approval from the Scientific Research Ethics Commission of the Victor Babeș University of Medicine and Pharmacy Timișoara (approval no. 11, dated 7 March 2024) and was registered at ClinicalTrials.gov (NCT06341569). The 12-month follow-up received separate approval from the same commission (approval no. 49, dated 9 May 2025). All procedures were conducted in accordance with institutional requirements and the principles of the Declaration of Helsinki (World Medical Association, 2013).

The follow-up assessment was conducted 12 months after program completion to evaluate long-term practice patterns and psychological outcomes. Participants completed the validated psychological instruments and the study-specific adherence questionnaire under standardized conditions. This study is reported in accordance with the Strengthening the Reporting of Observational Studies in Epidemiology (STROBE) guideline for cohort studies (von Elm et al., 2007).

### 2.2. Participants

The original study included 46 physiotherapy students: 34 in the structured breathing program group and 12 in the non-intervention control group (Crișan et al., 2025). The present longitudinal analysis was restricted to the 34 students who completed the breathing program because the primary follow-up objective concerned the maintenance of breathing practice after exposure to the intervention. All 34 intervention participants completed the 12-month assessment and were included in the analysis.

At T2, participants completed the study-specific Adherence Questionnaire and reported whether they currently practiced the breathing exercises learned during the program. Based exclusively on the practice-status response, participants reporting “regularly” or “occasionally” were classified in the ongoing-practice group (*n* = 18), whereas participants reporting “rarely” or “no” were classified in the no/rare-practice group (*n* = 16). Practice frequency and session duration were analyzed as separate adherence characteristics and were not used to define the two groups.

Practice status was measured at the 12-month assessment and was not assigned prospectively. Consequently, the two groups represent retrospectively identified practice patterns rather than intervention groups, and comparisons between them cannot establish that continued practice caused subsequent psychological differences.

### 2.3. Psychological Outcome Measures

#### 2.3.1. Subjective Happiness Scale (SHS)

The SHS is a four-item self-report instrument assessing global subjective happiness. Items are rated on a 7-point scale, with higher scores indicating greater subjective happiness. The fourth item is reverse-scored, and the final score is calculated as the mean of the four items, resulting in a possible score ranging from 1 to 7 (Lyubomirsky & Lepper, 1999).

#### 2.3.2. Oxford Happiness Questionnaire (OHQ)

The OHQ is a 29-item self-report instrument assessing broad aspects of psychological well-being and happiness. Items are rated on a 6-point scale ranging from 1 (“strongly disagree”) to 6 (“strongly agree”). Twelve negatively worded items are reverse-scored, and the final score is calculated as the mean of all 29 items, with higher scores indicating greater happiness (Hills & Argyle, 2002; Robbins et al., 2010).

#### 2.3.3. Positive and Negative Affect Schedule (PANAS)

The PANAS is a 20-item questionnaire designed to measure two separate dimensions of affect: positive affect (PA) and negative affect (NA). It includes two 10-item subscales, each assessing how much respondents have experienced specific emotions (e.g., “enthusiastic,” “distressed”) within a specific time frame (Watson et al., 1988). Items are rated on a 5-point Likert scale from “very slightly or not at all” to “extremely”. Higher scores on the PA subscale indicate higher levels of positive emotions and energy, while higher scores on the NA subscale indicate increased distress and negative engagement. The PANAS shows good internal consistency (Cronbach’s α usually 0.86–0.90 for PA and 0.84–0.87 for NA) and has been validated in various populations (Crawford & Henry, 2004).

#### 2.3.4. Pittsburgh Sleep Quality Index (PSQI)

The PSQI is a 19-item questionnaire designed to evaluate sleep quality and disturbances over the past month. It produces a global score based on seven components: subjective sleep quality, sleep latency, sleep duration, habitual sleep efficiency, sleep disturbances, use of sleep medication, and daytime dysfunction. Each component is scored from 0 to 3, and the total score ranges from 0 to 21, with higher scores indicating worse sleep quality (Buysse et al., 1989). A global score above 5 usually signifies clinically significant sleep disturbance. The PSQI has shown good internal consistency (Cronbach’s α ≈ 0.83) and strong validity in both clinical and non-clinical groups (Mollayeva et al., 2016).

#### 2.3.5. Penn State Worry Questionnaire (PSWQ)

The PSWQ is a 16-item self-report instrument designed to assess the frequency and intensity of trait worry. Items are rated on a 5-point scale ranging from 1 (“not at all typical of me”) to 5 (“very typical of me”). Five positively worded items are reverse-scored, and the item scores are summed to produce a total score ranging from 16 to 80, with higher scores indicating greater worry (Meyer et al., 1990). The PSWQ has demonstrated high internal consistency and established validity in clinical and non-clinical populations (Brown et al., 1992). Internal consistency was also excellent in the present sample at T2 (Cronbach’s α = 0.90).

#### 2.3.6. Satisfaction with Life Scale (SWLS)

The SWLS is a short self-report tool designed to measure overall life satisfaction, representing the cognitive part of subjective well-being. It includes 5 items scored on a 7-point Likert scale from 1 (“strongly disagree”) to 7 (“strongly agree”); no items are reverse-scored, and the total score ranges from 5 to 35 (Diener et al., 1985). Higher scores indicate greater life satisfaction, while lower scores suggest dissatisfaction. The scale has shown good internal consistency (Cronbach’s α ≈ 0.85) and strong convergent validity, negatively related to depression measures and positively related to health and well-being indicators (López-Ortega et al., 2016).

#### 2.3.7. Ryff Scales of Psychological Well-Being

The 42-item version of the Ryff Scales of Psychological Well-Being was used to assess six dimensions of psychological well-being: autonomy, environmental mastery, personal growth, positive relations with others, purpose in life, and self-acceptance. Each dimension comprises seven items rated on a 7-point Likert scale. Negatively keyed items were reverse-scored according to the published scoring instructions, and the item scores were summed separately for each dimension. Domain scores range from 7 to 49, with higher scores indicating greater psychological well-being (Abbott et al., 2006; Ryff & Keyes, 1995).

### 2.4. Adherence Questionnaire

A study-specific questionnaire was developed to characterize breathing-practice patterns at the 12-month follow-up. It assessed current practice status (“no,” “rarely,” “occasionally,” or “regularly”), weekly frequency (“none,” “less often,” “1–2 times,” “3–5 times,” or “daily”), session duration (<5, 5–10, 10–15, or >15 min), principal breathing technique, and usual practice timing. Additional items assessed perceived difficulty, technique clarity, perceived effects on stress, sleep, positivity, and worry, intention to continue, barriers, resources used, and adverse effects. Multiple-response barriers and resources were coded as separate binary variables. For the main analyses, participants reporting regular or occasional practice were classified as having ongoing practice, whereas those reporting rare or no practice were classified as having no/rare practice. Practice status, weekly frequency, and session duration were also retained as ordinal variables for exploratory correlation analyses. The questionnaire was designed for descriptive purposes and was not treated as a validated adherence scale or used to calculate a composite adherence score. The complete questionnaire and coding rules are publicly available on the Open Science Framework (DOI: 10.17605/OSF.IO/T9XAP).

### 2.5. Statistical Analysis

Analyses were conducted in Python 3.12.13 using NumPy 2.3.5, pandas 2.2.3, and SciPy 1.17.0. Ordinary least-squares estimation and HC3 heteroscedasticity-consistent covariance matrices were implemented directly using NumPy 2.3.5, while Benjamini–Hochberg false-discovery-rate adjustment was performed using SciPy 1.17.0; statsmodels was not used. Participants were linked across source files by normalized names, then assigned de-identified participant numbers. The resulting dataset contained 34 participants and 102 person-time observations. All linkage, scoring, and source-reconciliation decisions were retained in an audit trail.

Scale scores were calculated at each time point according to the published scoring instructions for each instrument. Reverse-scored items were recoded before calculating the SHS, OHQ, PSWQ, and Ryff domain scores (Abbott et al., 2006; Brown et al., 1992; Hills & Argyle, 2002; Lyubomirsky & Lepper, 1999; Meyer et al., 1990; Robbins et al., 2010; Ryff & Keyes, 1995). The T2 assessment was administered using paper questionnaires. Item-level responses were entered into an electronic dataset and checked against the original paper forms. All 13 psychological outcomes were included in the longitudinal analyses: SHS, OHQ, PANAS positive affect, PANAS negative affect, PSQI, PSWQ, the six Ryff psychological well-being domains, and SWLS.

The primary analysis used complete T1–T2 pairs for all 13 psychological outcomes. For each outcome, an ordinary least-squares model regressed the T2 score on the corresponding T1 score and a binary T2 practice indicator (ongoing versus no/rare practice). The group coefficient estimates the adjusted T2 difference in raw-score units. Heteroscedasticity-consistent HC3 standard errors and 95% confidence intervals were used. Two-sided *p*-values were corrected across the 13 primary outcomes using the Benjamini–Hochberg false-discovery-rate procedure. Hedges’ g was calculated from favorable T1–T2 change scores; changes in PANAS negative affect, PSQI, and PSWQ were multiplied by −1 so that positive values consistently favored ongoing practice. Robustness was evaluated with Welch tests, Mann–Whitney tests, and 100,000 label-permutation tests. The random seed was 20260814.

Adherence characteristics were summarized descriptively because practice group and the questionnaire characteristics were recorded concurrently and were not independent exposures. Exploratory Spearman correlations related ordinal practice status, weekly frequency, and session duration to favorable change in all 13 psychological outcomes; the false-discovery-rate correction was applied across these 39 correlations. No missing T1 or T2 outcome values occurred, and no imputation was performed.

## 3. Results

Baseline demographic, anthropometric, and psychological characteristics according to later T2 practice status are shown in Table 1.

In T1-adjusted models, the ongoing-practice group had more favorable T2 scores for subjective happiness (adjusted difference 1.02, 95% CI 0.55 to 1.49), Oxford happiness (0.91, 95% CI 0.67 to 1.14), PANAS positive affect (5.17, 95% CI 3.04 to 7.30), sleep quality (PSQI: −1.96, 95% CI −2.68 to −1.23), Ryff autonomy (7.72, 95% CI 4.96 to 10.49), environmental mastery (5.21, 95% CI 2.80 to 7.62), personal growth (6.99, 95% CI 4.17 to 9.82), positive relations (6.10, 95% CI 3.61 to 8.60), purpose in life (8.73, 95% CI 6.76 to 10.70), self-acceptance (5.89, 95% CI 3.41 to 8.38), and life satisfaction (5.74, 95% CI 3.68 to 7.80); all q < 0.001. However, the ongoing-practice group also had higher PSWQ scores, indicating greater worry (4.52, 95% CI 1.44 to 7.60; q = 0.006). PANAS negative affect did not differ between groups (−0.50, 95% CI −3.51 to 2.50; q = 0.735). Raw T1-to-T2 changes for all 13 outcomes are presented in Table 2, and model R^2^ values and residual standard deviations are reported in Supplementary Table S2. These associations should not be interpreted as effects of continued practice because group membership was self-selected and measured at T2.

Standardized between-group differences in favorable T1-to-T2 change are presented in Figure 1 for all 13 psychological outcomes.

Points show Hedges’ g and horizontal lines show 95% confidence intervals; positive values favor the ongoing-practice group. Changes in PANAS negative affect, PSQI, and PSWQ were multiplied by −1 so that positive values consistently represent more favorable change. Filled squares identify outcomes significant after Benjamini–Hochberg correction across all 13 outcomes. OHQ, Oxford Happiness Questionnaire; PANAS, Positive and Negative Affect Schedule; PSQI, Pittsburgh Sleep Quality Index; PSWQ, Penn State Worry Questionnaire; SHS, Subjective Happiness Scale; SWLS, Satisfaction with Life Scale; T1, immediately post-intervention assessment; T2, 12-month follow-up assessment.

Sensitivity analyses supported the primary findings. All outcomes except PANAS negative affect remained significant after false-discovery-rate correction in the Welch, Mann–Whitney, and 100,000-permutation analyses. For SHS, OHQ, PANAS positive affect, PSQI, all six Ryff domains, and SWLS, the direction of the differences favored the ongoing-practice group. For PSWQ, the direction of the difference favored the no/rare-practice group. PANAS negative affect remained non-significant across all sensitivity analyses.

At T2, 14 participants reported practicing regularly, 4 occasionally, 15 rarely, and 1 not at all. Of the 18 ongoing practitioners, 17 practiced at least three times weekly and all reported sessions lasting at least 5 min. Most no/rare practitioners reported practicing only when stressed and for less than 5 min. Perceived benefits, intention to continue, and technique clarity were more common among ongoing practitioners, whereas occasional adverse effects were more common in the no/rare group. These contrasts are descriptive because the characteristics and practice status were collected concurrently, and several characteristics were closely related to the reported practice category (Table 3).

In exploratory analyses across all 13 psychological outcomes, 35 of the 39 correlations remained significant after false-discovery-rate correction (Table S1). Higher practice status, weekly frequency, and session duration were associated with more favorable changes in SHS, OHQ, PANAS positive affect, PSQI, all six Ryff domains, and SWLS. For PSWQ, higher practice status and longer session duration were associated with less favorable change, whereas weekly frequency was not significantly associated with change after correction. None of the three adherence measures was significantly associated with change in PANAS negative affect. These adherence measures were highly interrelated and were collected concurrently with the T2 outcomes; consequently, the correlations do not establish dose–response relationships and may reflect common-method bias, reverse causation, or the group definition itself.

## 4. Discussion

This exploratory 12-month follow-up found that students reporting ongoing breathing practice had more favorable T2 scores for subjective happiness, general happiness, positive affect, sleep quality, all six dimensions of psychological well-being, and life satisfaction after adjustment for their corresponding T1 scores. In contrast, the ongoing-practice group had higher worry scores, while negative affect did not differ between the groups. The consistency across robust, nonparametric, and permutation analyses strengthens the statistical description of these associations, but it does not overcome the observational design or establish that continued practice caused the differences (Balban et al., 2023; Ma et al., 2017; Zaccaro et al., 2018).

Common-method bias represents an important alternative explanation for the observed associations. Practice status and psychological outcomes were self-reported by the same participants during the same T2 assessment session, creating susceptibility to common-method variance and response-consistency effects; participants who perceived themselves as maintaining the practice may also have reported their well-being more favorably. The large and broadly consistent differences across several conceptually related self-report outcomes may therefore reflect response consistency, social desirability, or a general positive self-evaluation in addition to any association with breathing practice. Moreover, the standardized between-group differences observed here were substantially larger than the small-to-moderate effects typically reported in breathwork trials (Fincham et al., 2023) and should not be interpreted as estimates of a causal treatment effect. Reverse causation is also plausible, because students who felt better may have been more likely to continue practicing. The unfavorable PSWQ result and the absence of a difference in PANAS negative affect indicate that this pattern was not uniform across all outcomes, but they do not eliminate the possibility of common-method bias.

The more favorable findings for happiness, positive affect, sleep quality, psychological well-being, and life satisfaction are broadly consistent with reports that structured breathing practices may influence psychological outcomes, but comparisons with prior intervention studies must be made with caution. The association between ongoing practice and higher worry scores further indicates that the observed pattern was not uniformly favorable. One possible explanation is selective continuation, whereby students experiencing persistent worry may have been more likely to continue using breathing exercises as a coping strategy, while the reduction in the no/rare-practice group may reflect natural fluctuation or regression to the mean. Practice after the supervised program was neither randomized nor objectively monitored, and the ongoing-practice group may have differed in motivation, perceived benefit, response tendencies, or other unmeasured characteristics. Accordingly, the primary findings may reflect continued practice, selective continuation, shared-method measurement, or a combination of these factors.

The adherence questionnaire provides a descriptive account of how breathing exercises were used following supervised instruction. Almost all participants with ongoing practice reported practicing at least three times weekly, with sessions lasting at least five minutes, and primarily used diaphragmatic or pursed-lip breathing. Conversely, participants in the no/rare-practice group usually reported sessions shorter than five minutes and practicing when stressed. Ongoing practitioners also reported more frequent use of supporting resources and rated the techniques as easier to understand. These patterns are compatible with routine practice and the use of supporting materials, but the cross-sectional questionnaire cannot establish whether either factor promoted continued practice (Huberty et al., 2019; Jessen et al., 2025; Regehr et al., 2013). Practice features, perceived benefits, and practice status were assessed simultaneously at T2 and should not be viewed as independent determinants of adherence or as causes of better psychological outcomes. Although occasional negative effects were more common in the no/rare-practice group, the questionnaire did not record the specific symptoms experienced; these effects therefore cannot be characterized more precisely.

The exploratory adherence correlations broadly paralleled the binary group results for happiness, positive affect, sleep quality, psychological well-being, and life satisfaction. However, this pattern was not uniform: greater reported practice was associated with less favorable PSWQ change, and no significant associations were observed for PANAS negative affect. Practice status, frequency, and duration were collected at T2 and were strongly interrelated; frequency and duration also contributed to the behavioral distinction between the groups. These analyses should therefore be interpreted as descriptive coherence rather than evidence of a biological dose–response relationship.

Controlled breathing may influence cardiorespiratory coupling, vagal modulation, heart rate variability, emotional regulation, and hypothalamic–pituitary–adrenal axis activity (Balaji et al., 2025; Jerath & Beveridge, 2020; Jerath et al., 2015; Vargas-Uricoechea et al., 2024; Zaccaro et al., 2018). However, these effects depend on respiratory rate, breathing-phase timing, breath holds, and breathing effort, none of which were measured in this follow-up. Consequently, the proposed physiological mechanisms could not be tested, and the absence of an association with negative affect cannot be attributed to any particular breathing pattern.

Behavioral mechanisms might also contribute to continued practice, although habit formation was not measured. Participants with ongoing practice most frequently reported noon or evening as their usual practice time, whereas no/rare practitioners commonly reported practicing when stressed or before sleep. This pattern is compatible with a distinction between scheduled and reactive practice, but the questionnaire recorded only usual timing and did not assess automaticity, environmental cues, reminders, or habit strength. Routine formation and behavioral anchoring should therefore be regarded as hypotheses for future testing rather than mechanisms demonstrated by this study (Bentley et al., 2023; Lally et al., 2010).

Self-efficacy may contribute to continued engagement, because successful use of a self-regulation strategy could strengthen confidence in managing internal states and encourage further practice (Bandura, 1977; Bentley et al., 2023). However, self-efficacy was not measured, and perceived benefits were assessed concurrently with adherence; therefore, this proposed mechanism remains hypothetical.

This study has several limitations. First, the sample comprised only 34 students from one physiotherapy cohort, limiting statistical power, precision, and generalizability. The small sample may have reduced the ability to detect smaller associations, particularly for negative affect, and may also have produced unstable or inflated coefficient and effect-size estimates. Although the primary models were deliberately parsimonious, with only the corresponding T1 score and practice-group indicator included as predictors, model stability cannot be assumed in such a limited sample, and some degree of overfitting remains possible. The magnitude and precision of the reported associations should therefore be interpreted cautiously and require confirmation in larger, adequately powered studies. Second, T2 practice status was self-selected, retrospectively reported, and measured concurrently with the outcomes; residual confounding, reverse causation, recall bias, and social-desirability bias are plausible. Third, no objective measures of practice, respiratory rate, technique fidelity, or physiological responses were available. In addition, because the original control group did not complete the T2 assessment, the present study functioned as a single-arm follow-up and could not compare the 12-month outcomes of program-exposed participants with those of a group that had remained entirely unexposed to the breathing program. The comparisons between the ongoing- and no/rare-practice groups therefore cannot distinguish associations related to continued practice from changes that might have occurred over time independently of exposure to the program. Fourth, the analysis was revised after data audit rather than prespecified before data collection. The findings should be considered hypothesis-generating and require prospective replication. The results support further prospective evaluation of breathing-practice maintenance in university well-being programs, particularly for positive affect, sleep quality, and life satisfaction. They do not justify clinical-effectiveness claims. Future multicenter controlled studies should prespecify scoring and analysis, define adherence prospectively, use standardized electronic data-capture procedures, retain an appropriate control group, and objectively record practice frequency, session duration, respiratory rate, breathing-phase timing, and technique fidelity. Randomized comparisons of different breathing protocols could clarify whether particular techniques produce greater or more sustained benefits. Incorporating physiological measures, such as heart rate variability or salivary cortisol, may also help investigate the mechanisms underlying psychological changes.

## 5. Conclusions

At 12 months, ongoing breathing practice was associated with more favorable subjective happiness, general happiness, positive affect, sleep quality, psychological well-being, and life satisfaction after adjustment for immediately post-intervention scores. However, ongoing practice was also associated with higher worry scores, while negative affect did not differ. These mixed findings were robust across several sensitivity analyses but remain observational and may partly reflect common-method bias, response consistency, reverse causation, or residual confounding. Prospective controlled studies using objective adherence measures and independently collected outcomes are needed.

## Figures and Tables

**Figure 1:**
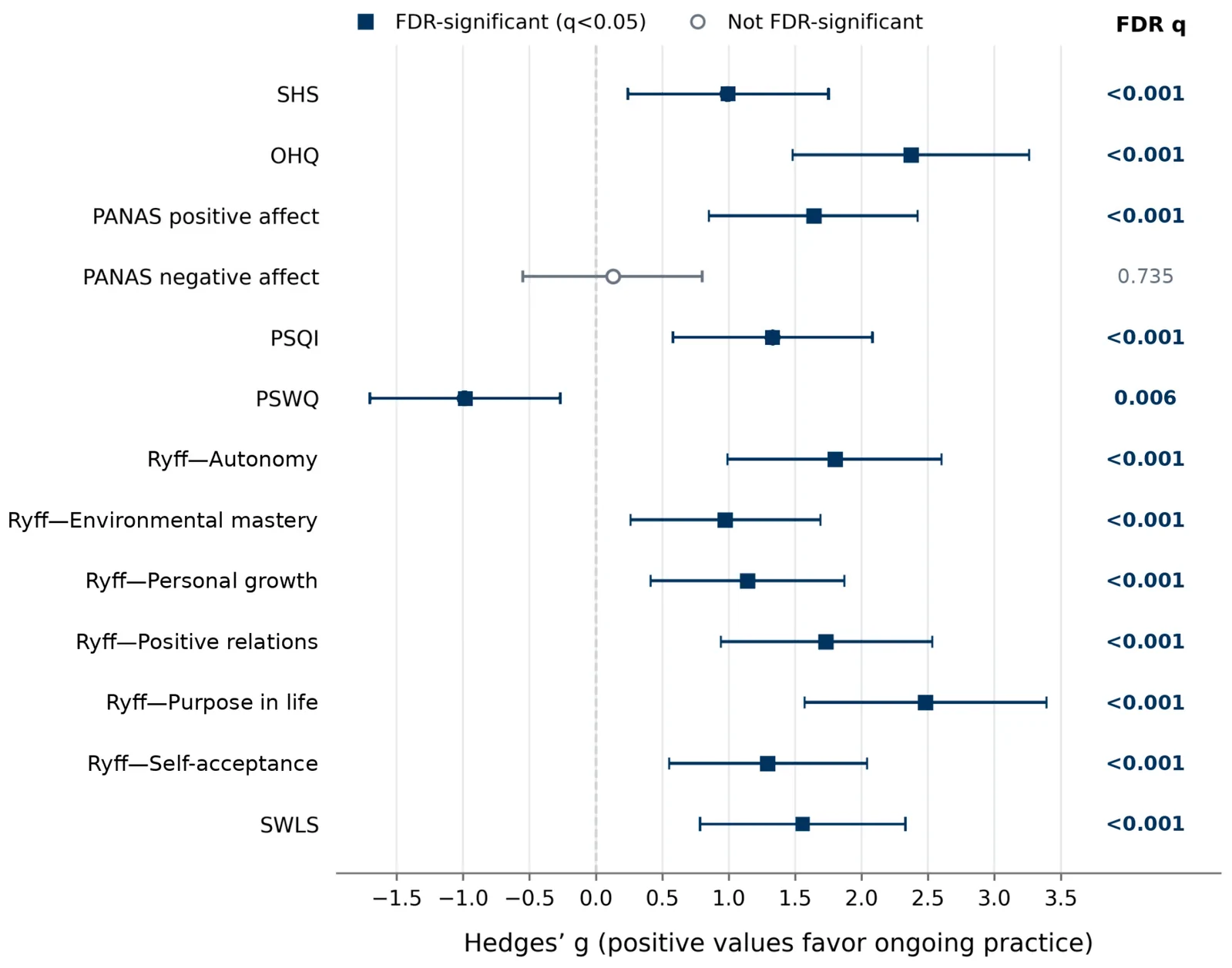
Standardized between-group differences in favorable T1-to-T2 change for all psychological outcomes.

**Table 1:** Baseline demographic, anthropometric, and psychological characteristics according to 12-month breathing-practice status.

Characteristic	Overall (*N* = 34)	No/Rare Practice (*n* = 16)	Ongoing Practice (*n* = 18)	SMD
Age, years	23.09 ± 6.88	22.44 ± 6.42	23.67 ± 7.40	0.18
Female sex, *n* (%)	20 (58.8%)	8 (50.0%)	12 (66.7%)	0.34
Height, cm	172.79 ± 10.45	172.75 ± 9.64	172.83 ± 11.40	0.01
Weight, kg	73.91 ± 19.83	71.19 ± 17.95	76.33 ± 21.57	0.26
SHS	4.38 ± 1.03	4.64 ± 1.10	4.15 ± 0.93	−0.48
OHQ	3.63 ± 0.34	3.63 ± 0.40	3.63 ± 0.29	0.02
PANAS positive affect	32.97 ± 5.75	33.44 ± 6.35	32.56 ± 5.32	−0.15
PANAS negative affect	23.24 ± 6.23	22.50 ± 6.23	23.89 ± 6.34	0.22
PSQI	6.79 ± 2.80	7.25 ± 2.89	6.39 ± 2.75	−0.30
PSWQ	47.21 ± 7.79	47.25 ± 6.69	47.17 ± 8.85	−0.01
Ryff autonomy	24.53 ± 4.49	24.56 ± 3.90	24.50 ± 5.08	−0.01
Ryff environmental mastery	26.32 ± 2.93	26.00 ± 2.61	26.61 ± 3.24	0.21
Ryff personal growth	28.79 ± 3.04	29.50 ± 3.12	28.17 ± 2.92	−0.44
Ryff positive relations	21.94 ± 3.57	22.56 ± 3.69	21.39 ± 3.47	−0.33
Ryff purpose in life	29.09 ± 4.42	29.50 ± 5.01	28.72 ± 3.94	−0.17
Ryff self-acceptance	24.06 ± 4.55	24.50 ± 3.83	23.67 ± 5.19	−0.18
SWLS	23.44 ± 5.12	22.56 ± 5.45	24.22 ± 4.83	0.32

Note: Data are mean ± standard deviation unless otherwise indicated. Psychological outcomes were assessed at T0, before the 14-week breathing program. SMDs compare ongoing with no/rare practice and are descriptive; positive values indicate higher scores in the ongoing-practice group. OHQ, Oxford Happiness Questionnaire; PANAS, Positive and Negative Affect Schedule; PSQI, Pittsburgh Sleep Quality Index; PSWQ, Penn State Worry Questionnaire; SHS, Subjective Happiness Scale; SMD, standardized mean difference; SWLS, Satisfaction with Life Scale; T0, pre-intervention assessment.

**Table 2:** T1-adjusted T2 differences for all psychological outcomes.

Outcome	No/Rare Practice T1 → T2	Ongoing Practice T1 → T2	Adjusted T2 Difference (95% CI)	FDR q
SHS	5.77 → 5.31 (Δ −0.45)	5.96 → 6.39 (Δ +0.43)	1.02 (0.55 to 1.49)	<0.001
OHQ	4.61 → 4.17 (Δ −0.43)	4.50 → 5.00 (Δ +0.50)	0.91 (0.67 to 1.14)	<0.001
PANAS positive affect	39.31 → 35.88 (Δ −3.44)	39.11 → 40.89 (Δ +1.78)	5.17 (3.04 to 7.30)	<0.001
PANAS negative affect	20.50 → 21.38 (Δ +0.88)	20.72 → 21.06 (Δ +0.33)	−0.50 (−3.51 to 2.50)	0.735
PSQI	4.75 → 5.50 (Δ +0.75)	4.44 → 3.39 (Δ −1.06)	−1.96 (−2.68 to −1.23)	<0.001
PSWQ	41.75 → 37.06 (Δ −4.69)	40.28 → 40.78 (Δ +0.50)	4.52 (1.44 to 7.60)	0.006
Ryff autonomy	28.88 → 24.50 (Δ −4.38)	26.56 → 31.44 (Δ +4.89)	7.72 (4.96 to 10.49)	<0.001
Ryff environmental mastery	26.38 → 20.81 (Δ −5.56)	26.94 → 26.17 (Δ −0.78)	5.21 (2.80 to 7.62)	<0.001
Ryff personal growth	28.94 → 25.19 (Δ −3.75)	31.44 → 32.83 (Δ +1.39)	6.99 (4.17 to 9.82)	<0.001
Ryff positive relations	23.88 → 22.12 (Δ −1.75)	20.94 → 27.78 (Δ +6.83)	6.10 (3.61 to 8.60)	<0.001
Ryff purpose in life	31.25 → 25.19 (Δ −6.06)	29.44 → 33.28 (Δ +3.83)	8.73 (6.76 to 10.70)	<0.001
Ryff self-acceptance	27.50 → 25.12 (Δ −2.38)	28.17 → 31.28 (Δ +3.11)	5.89 (3.41 to 8.38)	<0.001
SWLS	30.19 → 26.81 (Δ −3.38)	29.00 → 32.17 (Δ +3.17)	5.74 (3.68 to 7.80)	<0.001

Note: Adjusted differences are coefficients for ongoing versus no/rare practice from the T2 outcome models adjusted for the corresponding T1 score, using HC3 robust standard errors. FDR q-values were calculated using the Benjamini–Hochberg procedure across all 13 outcomes. The T1 and T2 values and raw changes shown in Table 2 remain on their original scales. For the calculation of Hedges’ g in Figure 1 only, change scores for PANAS negative affect, PSQI, and PSWQ were multiplied by −1 so that positive values consistently indicate more favorable change in the ongoing-practice group. Lower PANAS negative affect, PSQI, and PSWQ scores indicate more favorable outcomes. CI, confidence interval; FDR, false discovery rate; OHQ, Oxford Happiness Questionnaire; PANAS, Positive and Negative Affect Schedule; PSQI, Pittsburgh Sleep Quality Index; PSWQ, Penn State Worry Questionnaire; SHS, Subjective Happiness Scale; SWLS, Satisfaction with Life Scale; T1, immediately post-intervention assessment; T2, 12-month follow-up assessment.

**Table 3:** Selected adherence characteristics at the 12-month follow-up.

Characteristic	No/Rare Practice (*n* = 16)	Ongoing Practice (*n* = 18)
Practiced ≥3 times/week	0 (0.0%)	17 (94.4%)
Session duration ≥ 5 min	0 (0.0%)	18 (100.0%)
Diaphragmatic or pursed-lip breathing	0 (0.0%)	18 (100.0%)
Practiced only when stressed	11 (68.8%)	1 (5.6%)
Practice rated easy or very easy	2 (12.5%)	12 (66.7%)
Occasional adverse effects	9 (56.3%)	2 (11.1%)
Stress reduction rated ≥4/5	0 (0.0%)	18 (100.0%)
Sleep improvement rated ≥4/5	0 (0.0%)	15 (83.3%)
Increased positivity rated ≥4/5	0 (0.0%)	18 (100.0%)
Worry reduction rated ≥4/5	0 (0.0%)	14 (77.8%)
Intention to continue rated ≥4/5	0/15 (0.0%)	18/18 (100.0%)
Used no supporting resources	15 (93.8%)	0 (0.0%)

Note: Values are counts and within-group percentages. Practice groups and adherence characteristics were derived concurrently from the same 12-month questionnaire; therefore, the table is descriptive, and no between-group hypothesis tests were performed. One participant in the no/rare-practice group had an invalid intention-to-continue response. Adverse effects were reported as occasional negative effects, with questionnaire examples including dizziness or discomfort.

## Data Availability

The supporting data for the findings of this study can be obtained from the corresponding author upon reasonable request. The participant-level data cannot be made publicly available because of privacy and ethical restrictions. The adherence questionnaire and codebook are available on the Open Science Framework at DOI: 10.17605/OSF.IO/T9XAP.

## Supplementary Materials

The following supporting information can be downloaded at: https://www.mdpi.com/article/10.3390/bs16101745/s1, Table S1: Exploratory correlations between adherence measures and favorable T1-to-T2 changes in all 13 psychological outcomes; Table S2: Model fit statistics for T1-adjusted comparisons of T2 psychological outcomes.

## Abbreviations

The following abbreviations are used in this manuscript:

CI	Confidence interval
FDR	False discovery rate
HC3	Heteroscedasticity-consistent standard error estimator, type 3
OHQ	Oxford Happiness Questionnaire
PANAS	Positive and Negative Affect Schedule
PSQI	Pittsburgh Sleep Quality Index
PSWQ	Penn State Worry Questionnaire
SHS	Subjective Happiness Scale
SMD	Standardized mean difference
SWLS	Satisfaction with Life Scale
T0	Pre-intervention assessment
T1	Immediately post-intervention assessment
T2	12-month follow-up assessment
g	Hedges’ g effect size

## References

[B1-behavsci-16-01745] Abbott R. A., Ploubidis G. B., Huppert F. A., Kuh D., Wadsworth M. E. J., Croudace T. J. (2006). Psychometric evaluation and predictive validity of Ryff’s psychological well-being items in a UK birth cohort sample of women. Health and Quality of Life Outcomes.

[B2-behavsci-16-01745] Ahmed I., Hazell C. M., Edwards B., Glazebrook C., Davies E. B. (2023). A systematic review and meta-analysis of studies exploring prevalence of non-specific anxiety in undergraduate university students. BMC Psychiatry.

[B3-behavsci-16-01745] Balaji S., Plonka N., Atkinson M., Muthu M., Ragulskis M., Vainoras A., McCraty R. (2025). Heart rate variability biofeedback in a global study of the most common coherence frequencies and the impact of emotional states. Scientific Reports.

[B4-behavsci-16-01745] Balban M. Y., Neri E., Kogon M. M., Weed L., Nouriani B., Jo B., Holl G., Zeitzer J. M., Spiegel D., Huberman A. D. (2023). Brief structured respiration practices enhance mood and reduce physiological arousal. Cell Reports Medicine.

[B5-behavsci-16-01745] Bandura A. (1977). Self-efficacy: Toward a unifying theory of behavioral change. Psychological Review.

[B6-behavsci-16-01745] Bentley T. G. K., D’Andrea-Penna G., Rakic M., Arce N., LaFaille M., Berman R., Cooley K., Sprimont P. (2023). Breathing practices for stress and anxiety reduction: Conceptual framework of implementation guidelines based on a systematic review of the published literature. Brain Sciences.

[B7-behavsci-16-01745] Brown T. A., Antony M. M., Barlow D. H. (1992). Psychometric properties of the Penn State Worry Questionnaire in a clinical anxiety disorders sample. Behaviour Research and Therapy.

[B8-behavsci-16-01745] Buysse D. J., Reynolds C. F., Monk T. H., Berman S. R., Kupfer D. J. (1989). The Pittsburgh Sleep Quality Index: A new instrument for psychiatric practice and research. Psychiatry Research.

[B9-behavsci-16-01745] Crawford J. R., Henry J. D. (2004). The Positive and Negative Affect Schedule (PANAS): Construct validity, measurement properties and normative data in a large non-clinical sample. British Journal of Clinical Psychology.

[B10-behavsci-16-01745] Crișan A. F., Pescaru C. C., Maritescu A., Carunta V., Stoicescu E. R., Oancea C., Amăricăi E., Onofrei R. R. (2025). A 14-week structured breathing program: An investigation into its impact on psychological health parameters in university students. BMC Public Health.

[B11-behavsci-16-01745] Diener E., Emmons R. A., Larsen R. J., Griffin S. (1985). The Satisfaction With Life Scale. Journal of Personality Assessment.

[B12-behavsci-16-01745] Fincham G. W., Strauss C., Montero-Marin J., Cavanagh K. (2023). Effect of breathwork on stress and mental health: A meta-analysis of randomised-controlled trials. Scientific Reports.

[B13-behavsci-16-01745] Gambolò L., Pireddu R., D’Angelo M., Ticozzi E. M., Bellini L., Solla D., Fagoni N., Stirparo G. (2025). Exploring mental health of Italian college students: A systematic review and meta-analysis. Discover Mental Health.

[B14-behavsci-16-01745] Hills P., Argyle M. (2002). The Oxford Happiness Questionnaire: A compact scale for the measurement of psychological well-being. Personality and Individual Differences.

[B15-behavsci-16-01745] Huberty J., Green J., Glissmann C., Larkey L., Puzia M., Lee C. (2019). Efficacy of the mindfulness meditation mobile app “Calm” to reduce stress among college students: Randomized controlled trial. JMIR mHealth and uHealth.

[B16-behavsci-16-01745] Jerath R., Beveridge C. (2020). Respiratory rhythm, autonomic modulation, and the spectrum of emotions: The future of emotion recognition and modulation. Frontiers in Psychology.

[B17-behavsci-16-01745] Jerath R., Crawford M. W., Barnes V. A., Harden K. (2015). Self-regulation of breathing as a primary treatment for anxiety. Applied Psychophysiology and Biofeedback.

[B18-behavsci-16-01745] Jessen N. H., Løvschall C., Skejø S. D., Madsen L. S. S., Corazon S. S., Maribo T., Poulsen D. V. (2025). Effect of nature-based health interventions for individuals diagnosed with anxiety, depression and/or experiencing stress—A systematic review and meta-analysis. BMJ Open.

[B19-behavsci-16-01745] Lally P., van Jaarsveld C. H. M., Potts H. W. W., Wardle J. (2010). How are habits formed: Modelling habit formation in the real world. European Journal of Social Psychology.

[B20-behavsci-16-01745] López-Ortega M., Torres-Castro S., Rosas-Carrasco O. (2016). Psychometric properties of the Satisfaction with Life Scale (SWLS): Secondary analysis of the Mexican Health and Aging Study. Health and Quality of Life Outcomes.

[B21-behavsci-16-01745] Lyubomirsky S., Lepper H. S. (1999). A measure of subjective happiness: Preliminary reliability and construct validation. Social Indicators Research.

[B22-behavsci-16-01745] Ma X., Yue Z. Q., Gong Z. Q., Zhang H., Duan N. Y., Shi Y. T., Wei G. X., Li Y. F. (2017). The effect of diaphragmatic breathing on attention, negative affect and stress in healthy adults. Frontiers in Psychology.

[B23-behavsci-16-01745] Meyer T. J., Miller M. L., Metzger R. L., Borkovec T. D. (1990). Development and validation of the Penn State Worry Questionnaire. Behaviour Research and Therapy.

[B24-behavsci-16-01745] Mollayeva T., Thurairajah P., Burton K., Mollayeva S., Shapiro C. M., Colantonio A. (2016). The Pittsburgh Sleep Quality Index as a screening tool for sleep dysfunction in clinical and non-clinical samples: A systematic review and meta-analysis. Sleep Medicine Reviews.

[B25-behavsci-16-01745] Regehr C., Glancy D., Pitts A. (2013). Interventions to reduce stress in university students: A review and meta-analysis. Journal of Affective Disorders.

[B26-behavsci-16-01745] Robbins M., Francis L. J., Edwards B. (2010). Happiness as stable extraversion: Internal consistency reliability and construct validity of the Oxford Happiness Questionnaire among undergraduate students. Current Psychology.

[B27-behavsci-16-01745] Ryff C. D., Keyes C. L. M. (1995). The structure of psychological well-being revisited. Journal of Personality and Social Psychology.

[B28-behavsci-16-01745] Steinmane V., Fernate A. (2025). The effect of breathing exercises on adults’ sleep quality: An intervention that works. Frontiers in Sleep.

[B29-behavsci-16-01745] Tan G. X. D., Soh X. C., Hartanto A., Goh A. Y. H., Majeed N. M. (2023). Prevalence of anxiety in college and university students: An umbrella review. Journal of Affective Disorders Reports.

[B30-behavsci-16-01745] Vargas-Uricoechea H., Castellanos-Pinedo A., Urrego-Noguera K., Vargas-Sierra H. D., Pinzón-Fernández M. V., Barceló-Martínez E., Ramírez-Giraldo A. F. (2024). Mindfulness-based interventions and the hypothalamic–pituitary–adrenal axis: A systematic review. Neurology International.

[B31-behavsci-16-01745] von Elm E., Altman D. G., Egger M., Pocock S. J., Gøtzsche P. C., Vandenbroucke J. P. (2007). The Strengthening the Reporting of Observational Studies in Epidemiology (STROBE) statement: Guidelines for reporting observational studies. BMJ.

[B32-behavsci-16-01745] Watson D., Clark L. A., Tellegen A. (1988). Development and validation of brief measures of positive and negative affect: The PANAS scales. Journal of Personality and Social Psychology.

[B33-behavsci-16-01745] World Medical Association (2013). World Medical Association Declaration of Helsinki: Ethical principles for medical research involving human subjects. JAMA.

[B34-behavsci-16-01745] Zaccaro A., Piarulli A., Laurino M., Garbella E., Menicucci D., Neri B., Gemignani A. (2018). How breath-control can change your life: A systematic review on psycho-physiological correlates of slow breathing. Frontiers in Human Neuroscience.

