# Supplementary material for: Breathing-Practice Status and Psychological Outcomes 12 Months After a Structured Program in University Students
Source: Behav Sci (Basel). 2026 Sep 24;16(10):1745. doi: 10.3390/bs16101745 (PMC13701855; doi:10.3390/bs16101745)
Supplement: Supplementary file 1 [file behavsci-16-01745-s001.zip › behavsci-4533241-supplementary.pdf]

Table S1. Exploratory correlations between adherence measures and favorable T1–T2 changes in all 13 psychological outcomes.

Outcome	Ordinal practice status, ϱ (q)	Weekly frequency, ϱ (q)	Session duration, ϱ (q)
SHS	0.57 (<0.001)	0.53 (0.001)	0.53 (0.001)
OHQ	0.78 (<0.001)	0.77 (<0.001)	0.77 (<0.001)
PANAS positive affect	0.62 (<0.001)	0.57 (<0.001)	0.58 (<0.001)
PANAS negative affect	0.09 (0.638)	0.10 (0.608)	0.08 (0.644)
PSQI	0.63 (<0.001)	0.65 (<0.001)	0.60 (<0.001)
PSWQ	−0.40 (0.022)	−0.34 (0.056)	−0.38 (0.031)
Ryff autonomy	0.67 (<0.001)	0.65 (<0.001)	0.70 (<0.001)
Ryff environmental mastery	0.49 (0.004)	0.43 (0.013)	0.54 (0.001)
Ryff personal growth	0.59 (<0.001)	0.61 (<0.001)	0.54 (0.001)
Ryff positive relations	0.72 (<0.001)	0.69 (<0.001)	0.73 (<0.001)
Ryff purpose in life	0.74 (<0.001)	0.71 (<0.001)	0.76 (<0.001)
Ryff self-acceptance	0.56 (0.001)	0.55 (0.001)	0.55 (0.001)
SWLS	0.70 (<0.001)	0.73 (<0.001)	0.70 (<0.001)

Note: Values are Spearman's rank correlation coefficients (ϱ), with Benjamini–Hochberg-adjusted q-values in parentheses. The false-discovery-rate correction was applied across all 39 correlations (13 outcomes × three adherence measures). Favorable change was defined as T2 − T1 for SHS, OHQ, PANAS positive affect, the six Ryff dimensions, and SWLS, and as T1 − T2 for PANAS negative affect, PSQI, and PSWQ. OHQ, Oxford Happiness Questionnaire; PANAS, Positive and Negative Affect Schedule; PSQI, Pittsburgh Sleep Quality Index; PSWQ, Penn State Worry Questionnaire; SHS, Subjective Happiness Scale; SWLS, Satisfaction With Life Scale; T1, immediately post-intervention assessment; T2, 12-month follow-up assessment.

Table S2. Model fit statistics for T1-adjusted comparisons of T2 psychological outcomes.

Outcome	N	R^2	Residual SD
SHS	34	0.515	0.604
OHQ	34	0.779	0.340
PANAS positive affect	34	0.735	2.888
PANAS negative affect	34	0.452	4.217
PSQI	34	0.685	0.988
PSWQ	34	0.481	4.244
Ryff autonomy	34	0.571	3.533
Ryff environmental mastery	34	0.431	3.485
Ryff personal growth	34	0.590	3.436
Ryff positive relations	34	0.481	3.153
Ryff purpose in life	34	0.716	2.826
Ryff self-acceptance	34	0.565	3.203
SWLS	34	0.595	2.640

Note: Each model regressed the T2 outcome on the corresponding T1 score and practice group (ongoing versus no/rare practice). R^2 represents the proportion of

variance in the T2 outcome explained by the model. All models included 34 participants and had 31 residual degrees of freedom. HC3 robust standard errors were used for inference; this covariance adjustment does not alter the fitted values, R^2, or residual SD.
